# Clinical and radiological predictors of adverse outcomes after unilateral biportal endoscopic surgery for short-segment degenerative lumbar spinal stenosis

**DOI:** 10.3389/fsurg.2026.1860200

**Published:** 2026-08-26

**Authors:** Qiang Zhang, Ninghao Liu, Dawei Li, Yingjie Zheng

**Affiliations:** Department of Orthopedic Surgery, The Second Affiliated Hospital of Baotou Medical College, Baotou, China

**Keywords:** CT-based hounsfield unit, degenerative lumbar spinal stenosis, minimally invasive surgical procedures, postoperative complications, unilateral laminotomy for bilateral decompression

## Abstract

**Objective:**

Complications of lumbar spine stenosis surgery is not uncommon, and focusing on predicting complications following spinal endoscopic surgery remain limited. Our study aimed to collect clinical and imaging markers to identify risk factors for postoperative complications in spinal endoscopic surgery.

**Methods:**

Retrospective analysis was conducted on clinical and imaging data from patients undergoing unilateral laminotomy for bilateral decompression (ULBD) surgery to predict postoperative complications. Bone density was assessed using the CT Hounsfield unit measurement method. Univariate and multivariate analyses were performed to identify factors influencing postoperative complications, and clinical outcomes before and after UBE surgery were compared. Finally, the predictive validity of these factors was evaluated using receiver operating characteristic (ROC) curve analysis, providing a reliable assessment of their clinical application value.

**Results:**

This study included 166 patients who underwent short-level ULBD surgery, of whom 23 experienced postoperative complications. Compared with the non-complication group, patients in the complication group were older (71.35 ± 5.89 vs. 66.42 ± 8.27, *P* = 0.007) and had a longer duration of symptoms (26.09 ± 16.83 vs. 8.90 ± 3.87, *P* < 0.001). Multivariate analysis identified symptom duration exceeding one year as the sole independent risk factor for postoperative complications following ULBD surgery, with a prediction accuracy of 95.3%. Functional outcomes after UBE surgery were significantly improved compared with preoperative status in all patients.

**Conclusion:**

ULBD surgery demonstrates high efficacy in treating short-segment lumbar spinal stenosis. A symptom duration exceeding 15.55 months is an independent risk factor for postoperative complications following ULBD.

## Introduction

Degenerative lumbar spinal stenosis is estimated to have a prevalence of 9% in the general population and up to 47% in people older than 60 years (1, 2). Only small proportion of patients who have persistent symptoms and dysfunction despite nonoperative treatment are referred for spinal decompression surgery (3). Although there is emerging evidence that most patients improve after surgery, the key clinical and imaging factors that predict the final outcome remain uncertain.

Studies have reported that magnetic resonance imaging (MRI) can predict postoperative complications in patients with degenerative lumbar spinal stenosis (4). Similarly, CT-based Hounsfield unit (CT-HU) measurements can predict mechanical complications after internal fixation surgery, such as screw loosening and cage subsidence (5–7). However, these findings primarily pertain to the traditional posterior surgery.

In recent years, minimally invasive spine surgery (MISS) has preferred among spine surgeons due to its smaller incisions and faster postoperative recovery (8). Unilateral biportal endoscopic (UBE) surgery is a representative MISS technique and has demonstrated good efficacy in treating degenerative lumbar spinal stenosis (9). Nevertheless, UBE is also associated with its own adverse outcomes, such as postoperative cerebrospinal fluid leakage and nerve root injury (10) which are distinct from those seen in traditional surgery (11, 12). While some studies have focused on predicting specific adverse outcomes after UBE surgery, comprehensive prediction models covering all adverse outcomes within a single study cohort remain scarce. Therefore, this study aims to collect clinical and imaging data from patients with degenerative lumbar spinal stenosis undergoing unilateral laminotomy for bilateral decompression (ULBD) at our institution to develop a predictor for overall adverse outcomes.

## Materials and methods

### Patients population

A total of 166 consecutive patients who underwent ULBD for short segment degenerative lumbar spinal stenosis at our hospital between January 2022 and June 2025 were included in this study. All patients were followed up for a minimum of 6 months postoperatively. Ethical approval was granted by the local Institutional Review Board of the second affiliated hospital of Baotou medical college. The research conformed to the principles of the Declaration of Helsinki, with documented informed consent was obtained from all subjects and/or their legal guardian(s).

The inclusion criteria were: 1) patients who underwent ULBD for short segment (1–2 segment) degenerative lumbar spinal stenosis and failed conservative treatment; 2) with complete preoperative and postoperative imaging data including plain x-ray pictures (anteroposterior, lateral, flexion, and extension views), Computed Tomography (CT) scanning, magnetic resonance imaging (MRI) available at our institution. Each patient was followed up for at least 6 months after surgery and used Clavien Dindo classification (CDC) to evaluate the grading of complications (13). Conversely, the exclusion criteria were: 1) patients with lumbar spine trauma, neoplastic lesions, infectious pathologies, congenital malformations, or systemic disorders (including rheumatoid arthritis and neurodegenerative diseases) that could compromise surgical outcomes; (2) prior lumbar spine surgical interventions; (3) insufficient follow-up data or documented history of substance use disorders.

### Surgical technique

After the induction of general anesthesia, the patient was placed in the prone position. A biportal endoscopic unilateral laminotomy for bilateral decompression (BE-ULBD) was performed via the inter-laminar approach (14, 15). Sequential dilation was carried out using metal tubes of increasing diameter, with the working channel positioned at the junction between the inferior border of the lamina and the spinous process. The final instrument position was confirmed using C-arm. Under endoscopic visualization, anatomical structures were progressively exposed, including the inferior edge of the contralateral lamina, the spinous process, the facet joints, and the superior border of the lamina. A partial laminectomy was then performed by removing the inferior portion and superior margin of the lamina. The lamina was drilled along the butterfly-shaped attachment of the ligamentum flavum to adequately expose the nerve roots. During contralateral “over-the-top” decompression, the contra-lateral ligamentum flavum was resected. Finally, a drainage tube was placed, and the incision was closed in layers and covered with sterile dressing, thus completing the procedure. If radiological findings reveal significant lumbar instability, percutaneous pedicle screw fixation is subsequently performed (16).

### Clinical outcome measures

Retrospectively collected data included patient demographics (age, gender, BMI), lifestyle factors (smoking, drinking), symptom duration, previous illnesses (hypertension, diabetes), surgical details (segment, blood loss, operative time), and perioperative complications. Standardized follow-up assessments involved clinical and imaging evaluations at scheduled intervals at least 6 months, with outcomes measured using the ODI and VAS score for function and pain.

### Radiological assessments

**Figure 1:**
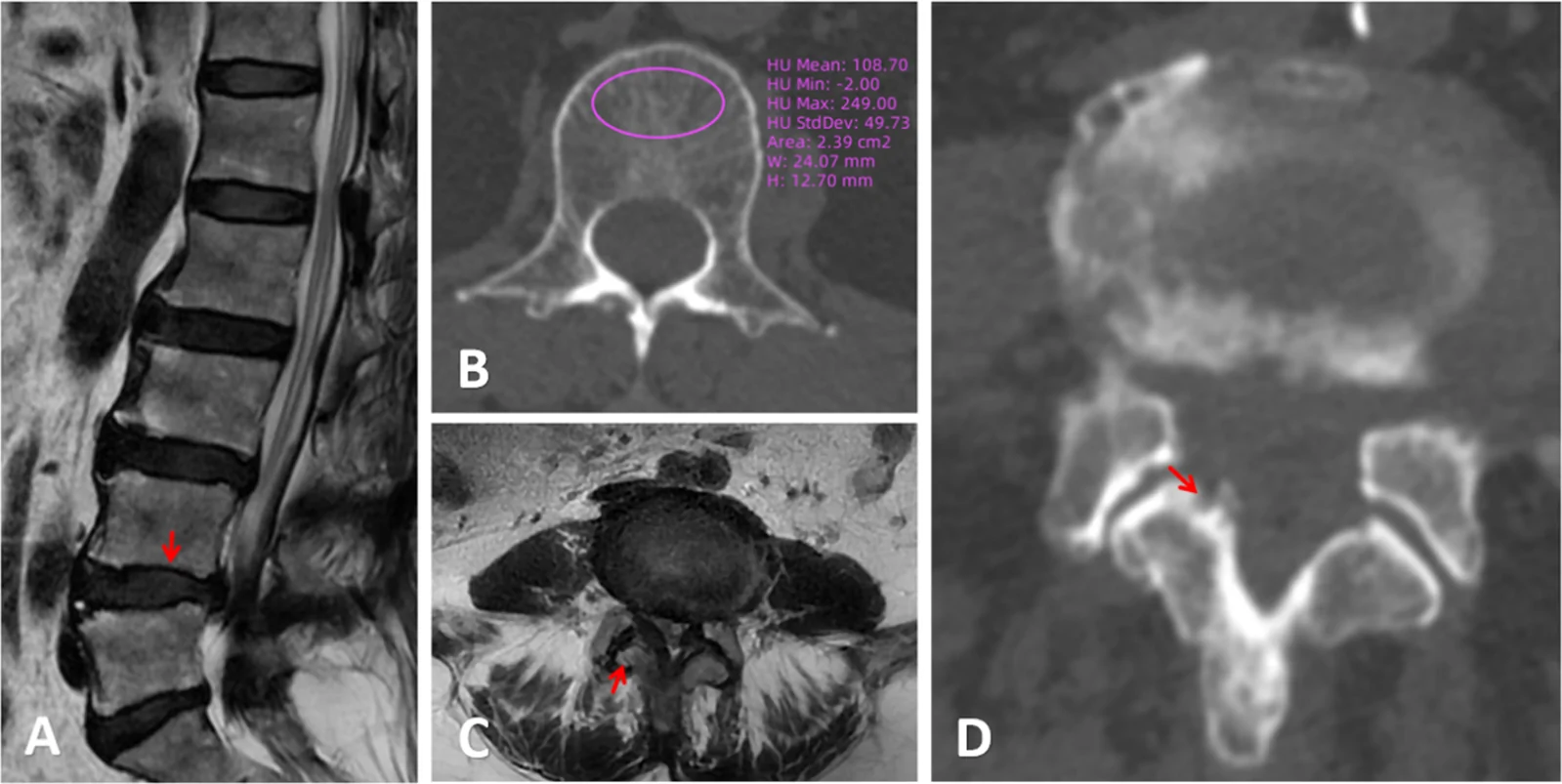
**(A)** T2-weighted MRI shows grade IV degeneration of the L4-5 intervertebral disc. **(B)** Axial CT image at the mid-portion of the L1 vertebral body shows an anterior column CT-HU value of 108.70. **(C)** Axial MRI reveals severe stenosis at the affected level, with grade III facet joint degeneration. **(D)** The facet joints at the affected level are classified as grade III.

Preoperative standing anteroposterior, lateral, flexion, and extension x-rays were obtained to assess lumbar stability. Preoperative CT scans were used to characterize the nature of the compressive pathology, the degree of facet joint degeneration and the condition of osteoporosis (17–19), while preoperative MRI was performed to evaluate the severity of stenosis and degree of intervertebral disc degeneration (Figure 1) (20, 21). The measurement method of CT-HU is derived from the article by Zou et, al (22). Intraoperative spinal endoscopic visualization allowed direct assessment of decompression adequacy. At the final postoperative follow-up, x-ray, CT, and MRI were repeated to evaluate the extent of spinal canal expansion.

### Statistical analysis

All statistical procedures were conducted in SPSS (version 26; IBM Corp., Armonk, NY) with a predetermined significance threshold of *P* < 0.05 (two-tailed). Descriptive statistics for continuous variables are reported as the mean ± standard deviation, with group comparisons conducted via the Student's t-test. Categorical data are summarized as frequencies and proportions and were analyzed using the Pearson's chi-square test. Logistic regression analysis was performed to determine the independent risk factors. Receiver operating characteristic (ROC) curve analysis was conducted to assess the diagnostic performance, by which the optimal cutoff value was determined using the Youden index for a rigorous evaluation of the model's discriminative ability.

## Results

### Analysis of imaging factors for adverse complications

This study retrospectively analyzed 166 patients diagnosed with short-segment lumbar spinal stenosis who underwent ULBD at our institution. Among the study cohort, 4 patients underwent internal fixation surgery due to lumbar instability. Postoperative complications occurred in 23 patients (13.86%), including nerve root injury (*n* = 2, CDC II), dural tear (*n* = 5, CDC I), superficial surgical site infection (*n* = 3, CDC I), spinal hypertension-like syndrome (*n* = 1, CDC IV), foot drop (*n* = 1, CDC II), atypical postoperative pain (*n* = 4, CDC II), iatrogenic instability-induced low back pain (*n* = 2, CDC II), inadequate decompression (*n* = 3, CDC II), epidural hematoma (*n* = 1, CDC I), and distal cerebellar hemorrhage (*n* = 1, CDC V). The 23 patients with complications were assigned to the adverse group (AG), while the remaining patients constituted Non-adverse group (Non-AG). The baseline characteristics of both groups are presented in Table 1. Significant inter-group differences were observed with regard to age (71.35 ± 5.89 vs. 66.42 ± 8.27, *P* = 0.007), symptom duration (26.09 ± 16.83 vs. 8.90 ± 3.87, *P* < 0.001), while the remaining demographic and clinical variables showed no statistically significant disparities. The median, maximum, and minimum duration of symptoms in the complication group are respectively 18, 11,and 74 months, and the duration of symptoms in the group without complications are respectively 9, 2 and 24 months.

**Table 1:** Patients demographic baseline data of ULBD.

Patients characteristics	Adverse group (*n* = 23)	Non-Adverse group (*n* = 143)	*P-*value
Age (Years)	71.35 ± 5.89	66.42 ± 8.27	**0**.**007**
Males	13/23	74/143	0.671
BMI (Kg/m^2^)	24.14 ± 1.48	24.66 ± 3.24	0.215
Smoking	9/23	52/143	0.798
Alcoholism	6/23	40/143	0.851
Hypertension	8/23	55/143	0.736
Cardiovascular disease	3/23	25/143	0.598
Diabetes	5/23	41/143	0.491
Duration of symptom	26.09 ± 16.83	8.90 ± 3.87	**<0**.**001**
VAS (leg)	5.96 ± 1.07	6.25 ± 0.99	0.190
VAS (Lumbar)	4.35 ± 1.07	4.43 ± 0.98	0.700
ODI	66.70 ± 5.68	65.52 ± 5.21	0.324
CSA of dural sac (mm^2^)	69.35 ± 11.05	66.57 ± 11.68	0.288
Pfirrmann Grade (≥3)	22/23	116/143	0.084
Weishaupt Grade (≥2)	5/23	29/143	0.872
CT-HU (≤115)	16/23	71/143	0.076
Operational time (min)	12.22 ± 4.18	11.01 ± 6.16	0.365
Duration of symptom (exceeding 12 months)	20/23	25/143	**<0**.**001**

Bold values indicate statistically significant differences.

Variables with a *P* value < 0.1 in the univariate analysis were subsequently entered into the logistic regression model. This analysis identified symptom duration as the sole independent risk factor for adverse outcomes (odds ratio [OR] = 1.837, 95% CI = [1.401–2.409], *P* < 0.001) shown as Table 2.

**Table 2:** Multivariate logistic regression analysis for risk factors of adverse outcomes after ULBD.

Variable	Odds Ratio	95% CI	*P-*value
Age	1.115	0.963–1.291	0.147
Duration of symptom	1.837	1.401–2.409	**<0**.**001**
Pfirrmann classification (≥3 Grade)	0.283	0.007–11.856	0.508
CT-HU (≤115)	6.097	0.604–61.595	0.126
Duration of symptom (exceeding 12 months)	31.467	8.680–114.067	**<0**.**001**

Bold values indicate statistically significant differences.

**Figure 2:**
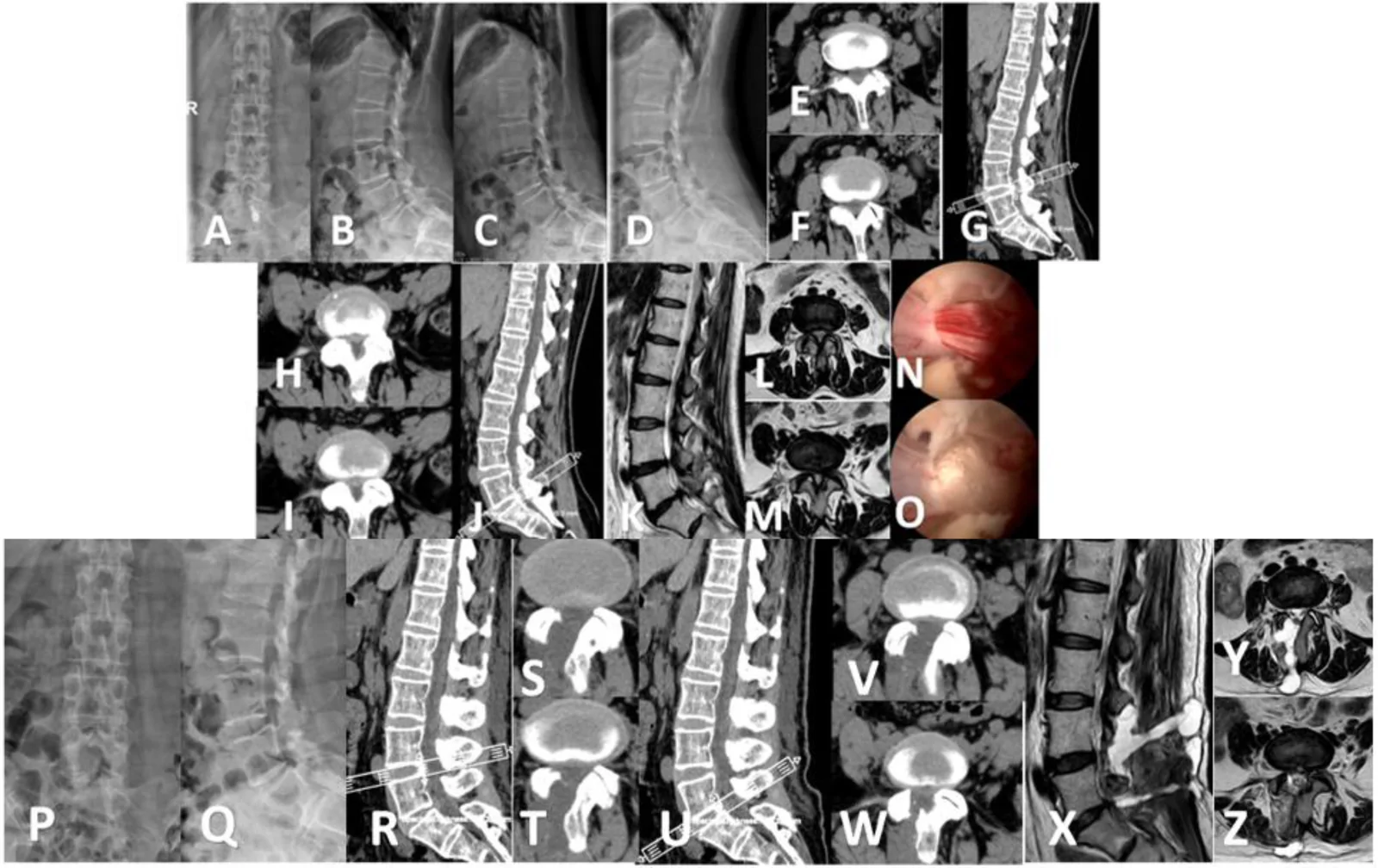
**(A–D)** preoperative lumbar spine anteroposterior, lateral, and flexion-extension views showed no significant instability. **(E–J)** Preoperative lumbar spine CT demonstrated lumbar spinal stenosis at L4–S1 without osseous protrusion. **(K–M)** Preoperative MRI revealed severe stenosis and soft disc protrusion at L4–S1. **(N,O)** Intraoperatively, dural tear was observed and was packed with gelatin sponge. **(P,Q)** Postoperative lumbar spine anteroposterior and lateral views showed no evidence of instability. **(R–Z)** Postoperative CT and MRI demonstrated adequate decompression with evidence of cerebrospinal fluid leakage.

Furthermore, the analysis of clinical efficacy demonstrated that ULBD surgery significantly improved patients' quality of life, as evidenced by the substantial improvements in VAS (leg), VAS (lumbar) and ODI scores from pre-operation (6.21 ± 1.00, 4.42 ± 0.97, 65.69 ± 5.27) to the final follow-up (0.90 ± 0.66, 1.37 ± 0.56, 13.72 ± 2.64, *P* < 0.001). Postoperative imaging revealed a significant increase in the cross-sectional area of the stenosis segments compared to preoperative values shown as Table 3 (140.39 ± 15.22 vs. 66.96 ± 11.60, *P* < 0.001). To assess its predictive value, a ROC curve analysis was performed for symptom duration. The area under the curve (AUC) was calculated, and the Youden index was determined, as illustrated in Figure 2.

**Table 3:** Comparison of clinical and imaging parameters after ULBD.

Variable	VAS (leg)	VAS(lumbar)	ODI	CSA
Pre-operation	6.21 ± 1.00	4.42 ± 0.97	65.69 ± 5.27	66.96 ± 11.60
Post-operation	0.90 ± 0.66	1.37 ± 0.56	13.72 ± 2.64	140.39 ± 15.22
*P* value	**<0.001**	**<0.001**	**<0.001**	**<0.001**

Bold values indicate statistically significant differences.

## Cases report

### Case 1

**Figure 3:**
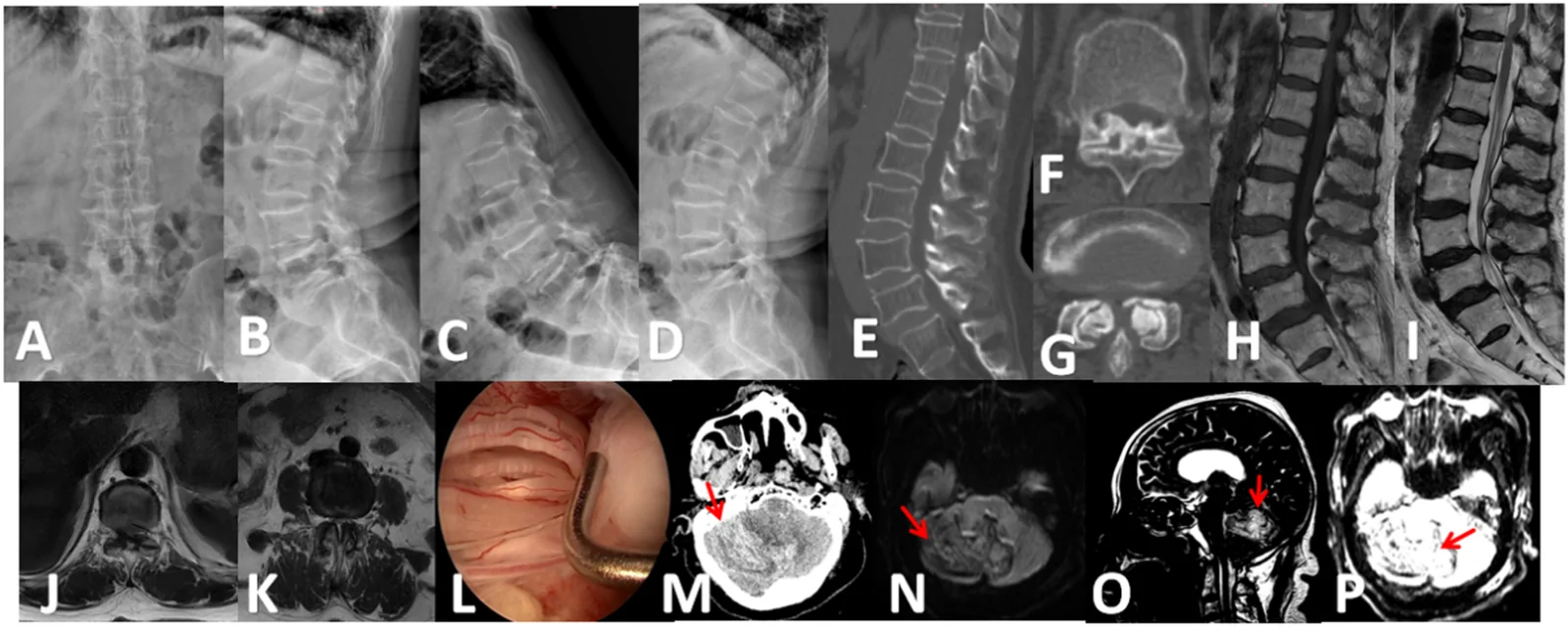
**(A–D)** preoperative lumbar spine anteroposterior, lateral, and flexion-extension views showed no significant instability. **(E–G)** Preoperative lumbar spine CT revealed ossification of the ligamentum flavum at T11–T12 and L4–L5, resulting in spinal canal stenosis. **(H–K)** Preoperative MRI indicated severe spinal canal stenosis at T11–T12 and L4–L5. **(L)** Intraoperatively, a dural tear was observed. **(M–P)** Postoperative cranial CT and MRI demonstrated extensive hemorrhage in the distal cerebellum.

A 52-year-old female presented with symptomatic L4-5 and L5-S1 spinal stenosis, as confirmed by both clinical examination and preoperative imaging. She underwent the ULBD surgery. The procedure was complicated by an intraoperative dural tear, which was managed with a gelatin sponge. Following extubation, the patient developed acute spinal hypertension-like syndrome, necessitating emergency reintubation and transfer to the intensive care unit. She recovered neurologically and was transferred back to the general ward the next day in a stable condition. However, her postoperative course was subsequently prolonged due to delayed mobilization secondary to the dural tear (Figure 3). These images have been obtained with the informed consent of the patients.

### Case 2

**Figure 4:**
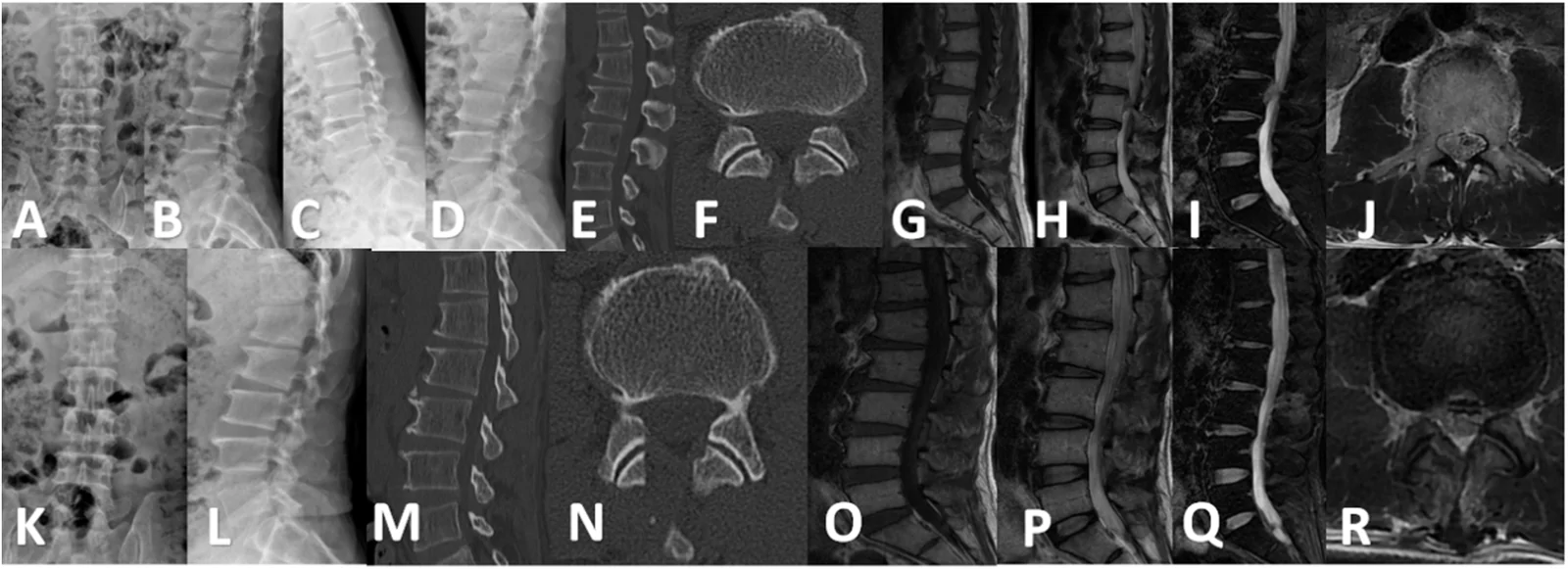
**(A–D)** preoperative lumbar spine anteroposterior, lateral, and flexion-extension views showed no significant instability. **(E,F)** Preoperative CT revealed no significant osseous compression. **(G–J)** Preoperative MRI indicated spinal canal stenosis due to soft disc compression at L2–L3. **(K,L)** Postoperative anteroposterior and lateral views showed no significant iatrogenic instability. **(M,N)** Postoperative CT demonstrated adequate spinal canal decompression. **(O–R)** Postoperative MRI showed that the soft disc protrusion had been removed, with satisfactory spinal canal decompression.

A 65-year-old male with a preoperative diagnosis of T12-L1 and L4-5 spinal stenosis underwent ULBD at our hospital. The procedure was complicated by an intraoperative dural tear. Two days postoperatively, the patient experienced sudden onset vomiting and limb numbness. Emergency head CT and MRI revealed distal cerebellar hemorrhage. Despite maximal resuscitation efforts, the patient succumbed to the hemorrhage and detailed findings are presented in Figure 4. These images have been obtained with the informed consent of the patients.

### Case 3

**Figure 5:**
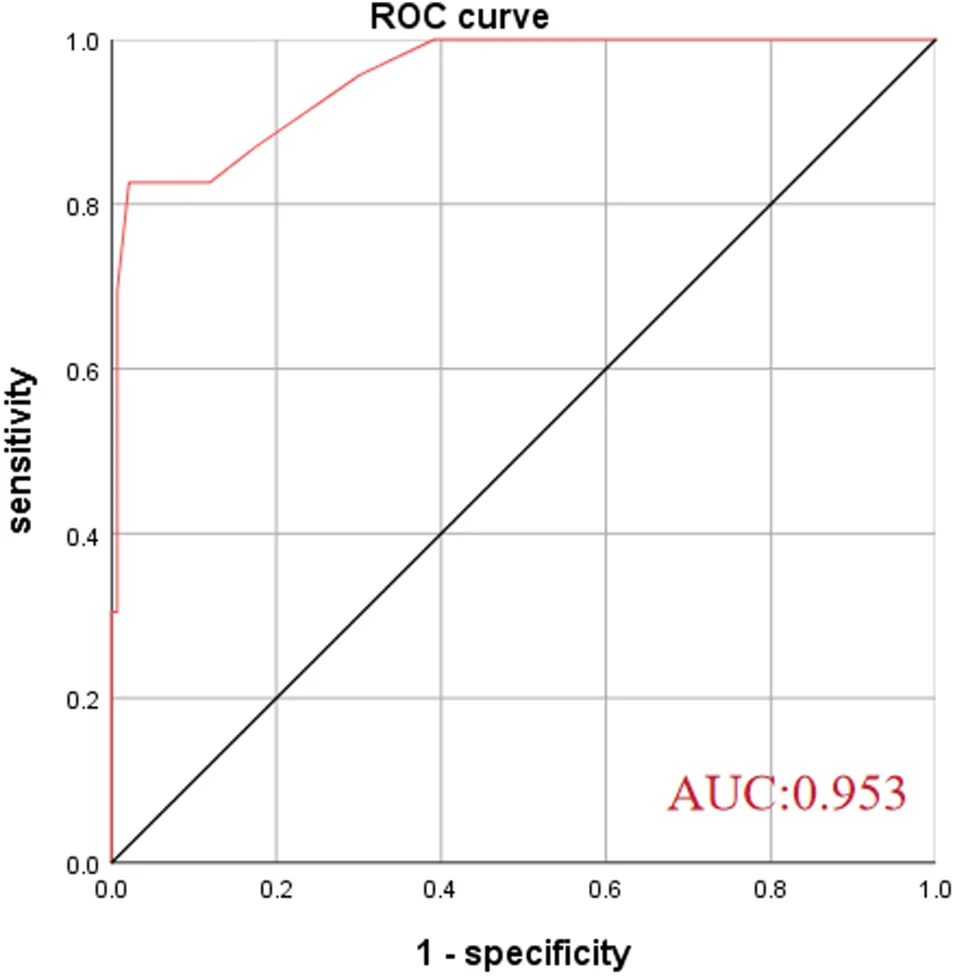
The area under the curve (AUC) was 0.953 (95% CI: 0.912–0.995). The optimal cutoff value determined by the Youden index was 15.55, yielding a sensitivity of 82.6% and a specificity of 97.9%.

A 48-year-old male with a preoperative diagnosis of L2-L3 spinal stenosis and lumbar disc herniation underwent ULBD and discectomy at our hospital. Postoperatively, the patient developed foot drop, as illustrated in Figure 5. These images have been obtained with the informed consent of the patients.

## Discussion

The adverse outcomes after lumbar spine surgery range from common and temporary problems to serious and permanent complications. These include complications such as surgical site infection, dural tear, epidural hematoma, and nerve injury (23, 24). Systemic complications such as venous thromboembolism, pulmonary, gastrointestinal, neurological, hematological, urologic complications and adverse cardiac events may also occur (25). In addition to immediate perioperative risks, adverse outcomes also include unconventional discharge, hospital readmission, revision surgery, and “failed lumbar spine surgery syndrome” where pain or function has not improved clinically (26). Numerous risk factors predispose patients to these poor outcomes. Patient-specific factors include advanced age, high body mass index, female patient, and surgical factors involve surgical procedures, and longer procedural times (27–29). Additionally, psychosocial elements are consistently linked to sub-optimal functional recovery and persistent disability postoperatively (30). In this study, we only analyzed postoperative complications associated with the UBE surgical approach, and the results showed that symptom duration was the sole independent risk factor for complications.

Spinal cord hypertension is a serious complication of endoscopic spine surgery for example UBE technique, caused by continuous irrigation fluid accumulating within a confined spinal compartment—such as the epidural or subdural space—due to impaired outflow (31). This results in elevated intraspinal pressure, compressing the thecal sac and neural structures. Clinical manifestations include severe headache, nausea, bradycardia, hypertension, altered consciousness, and progressive neurological deficits. If unrecognized, permanent neurological injury may occur. Risk factors include prolonged surgery, high irrigation pressure, inadequate outflow, and pre-existing dural defects. Prompt recognition, immediate reduction of irrigation pressure, and surgical decompression are essential to prevent irreversible damage.

Remote cerebellar hemorrhage (RCH) is a rare but potentially devastating complication following uniportal or biportal endoscopic (UBE) spine surgery (32, 33). Unlike direct surgical site bleeding, RCH occurs in the posterior fossa, typically involving the cerebellar hemispheres or vermis, at a location distant from the operative field. The most widely accepted mechanism is excessive intraoperative cerebrospinal fluid (CSF) loss—often due to incidental durotomy—leading to cerebellar sagging, stretching of supratentorial bridging veins, and subsequent venous infarction or hemorrhage. Clinical presentation usually manifests within 24 to 72 h postoperatively, with symptoms ranging from headache, nausea, and altered mental status to progressive neurological deterioration. Risk factors include intraoperative dural tear, prolonged surgery, and aggressive fluid drainage. Early recognition through prompt imaging is critical, as timely intervention can prevent permanent neurological sequelae.

Foot drop is a rare yet clinically significant complication following spinal surgery, particularly in the lumbar and sacral regions (34). Risk factors include increased intervertebral space tension, deformity correction, prolonged operative time, and advanced patient age. The primary mechanisms underlying postoperative foot drop encompass direct nerve trauma, tension- or stretch-induced injury, compression from hematoma or implants, and ischemic injury due to disrupted blood flow (35). Key strategies to mitigate the risk of foot drop include thorough preoperative consultation, meticulous intraoperative technique, appropriate management of intervertebral space tension, and cautious handling of nerve tension (36). Ongoing research is essential to establish standardized prevention and management guidelines. Treatment should be individualized based on the underlying etiology, ranging from conservative approaches such as physical therapy, ankle-foot orthotics, and functional electrical stimulation to surgical interventions including hematoma evacuation, implant removal, nerve decompression, nerve transfer, and tendon transfer. However, significant variability in clinical outcomes persists (37).

Several limitations should be taken into account in relation to our study. First, as a retrospective study, not all imaging data were available for collection, resulting in the loss of a portion of the sample. In addition, the small sample size and single-center design limit the reliability of our findings, and multi-center studies with larger cohorts are needed for validation. Additionally, although dual-energy x-ray absorptiometry is the gold standard for assessing osteoporosis, not all patients undergoing spinal surgery were able to undergo bone mineral density testing. Finally, this study did not evaluate patients' psychological factors, which we plan to assess in future research.

## Conclusion

The findings indicate that a symptom duration exceeding 15.55 months is an independent risk factor for postoperative complications in patients with lumbar spinal stenosis. Although severe postoperative complications are primarily associated with dural tears, further research is warranted to elucidate the specific relationship between symptom duration and such complications, and to inform the development of preventive strategies. Notably, unilateral laminotomy for bilateral decompression (ULBD) has been proven to be an effective minimally invasive technique that significantly alleviates symptoms of lumbar spinal stenosis, offering patients a reliable treatment option with favorable clinical outcomes.

## Data Availability

The raw data supporting the conclusions of this article will be made available by the authors, without undue reservation.

## References

[1] Anderson DB Beard DJ Rannou F Hunter DJ Suri P Chen L. Clinical assessment and management of lumbar spinal stenosis: clinical dilemmas and considerations for surgical referral. Lancet Rheumatol. (2024) 6(10):e727–32. 10.1016/S2665-9913(24)00028-6 38723654

[2] Tomkins-Lane C Melloh M Lurie J Smuck M Battié MC Freeman B. ISSLS prize winner: consensus on the clinical diagnosis of lumbar spinal stenosis: results of an international delphi study. Spine (Phila Pa 1976). (2016) 41(15):1239–46. 10.1097/BRS.0000000000001476 26839989 PMC4966995

[3] Katz JN Zimmerman ZE Mass H Makhni MC. Diagnosis and management of lumbar spinal stenosis: a review. JAMA. (2022) 327(17):1688–99. 10.1001/jama.2022.5921 35503342

[4] Caprariu R Oprea MD Poenaru DV Andrei D. Correlation between preoperative MRI parameters and oswestry disability Index in patients with lumbar spinal stenosis: a retrospective study. Medicina (B Aires). (2023) 59(11):2000. 10.3390/medicina59112000 PMC10672893 38004049

[5] Purnomo AE Tobing JFL Kurniawan AN Elbert R Heriyanto RS Arjuna YYE. Accuracy of CT hounsfield units for predicting cage subsidence and pedicle screw loosening after lumbar interbody fusion: a systematic review and meta-analysis. Neurosurg Rev. (2025) 48(1):602. 10.1007/s10143-025-03741-5 40815382

[6] Yao YC Chao H Kao KY Lin HH Wang ST Chang MC. CT Hounsfield unit is a reliable parameter for screws loosening or cages subsidence in minimally invasive transforaminal lumbar interbody fusion. Sci Rep. (2023) 13(1):1620. 10.1038/s41598-023-28555-7 36709341 PMC9884280

[7] Narayanan R Tarawneh OH Trenchfield D Meade MH Lee Y Opara O. Preoperative hounsfield units predict pedicle screw loosening in osteoporotic patients following short-segment lumbar fusion. Spine (Phila Pa 1976). (2024) 49(24):1722–8. 10.1097/BRS.0000000000004995 38556736

[8] Ding H Han X Xing Y Liu Y He D Han X. Clinical and radiological comparison of unilateral biportal endoscopic and percutaneous transforaminal endoscopic discectomy in the treatment of lumbar spinal degenerative disease. Orthop Surg. (2025) 17(4):1105–13. 10.1111/os.14361 39854041 PMC11962286

[9] Dou C Yu Q Zhang W Ma L Meng X. Comparison of biportal endoscopic technique and conventional unilateral laminectomy for bilateral decompression (ULBD) for multi-level degenerative lumbar spinal stenosis in elderly people. Orthop Surg. (2025) 17(8):2302–12. 10.1111/os.70084 40528425 PMC12318692

[10] Kalanchiam GP Kaliya-Perumal AK Sampath L Boey ETH Oh JY. Complications in uniportal vs unilateral biportal endoscopic decompression for lumbar spinal stenosis: a scoping review. Global Spine J. (2026) 16(1):724–33. 10.1177/21925682251346413 40448305 PMC12126473

[11] Shamji MF Mroz T Hsu W Chutkan N. Management of degenerative lumbar spinal stenosis in the elderly. Neurosurgery. (2015) 77(Suppl 4):S68–74. 10.1227/NEU.0000000000000943 26378360

[12] Wang B He P Liu X Wu Z Xu B. Complications of unilateral biportal endoscopic spinal surgery for lumbar spinal stenosis: a systematic review of the literature and meta-analysis of single-arm studies. Orthop Surg. (2023) 15(1):3–15. 10.1111/os.13437 36394088 PMC9837251

[13] Dindo D Demartines N Clavien PA. Classification of surgical complications: a new proposal with evaluation in a cohort of 6336 patients and results of a survey. Ann Surg. (2004) 240(2):205–13. 10.1097/01.sla.0000133083.54934.ae 15273542 PMC1360123

[14] Kim JE Choi DJ. Unilateral biportal endoscopic decompression by 30° endoscopy in lumbar spinal stenosis: technical note and preliminary report. J Orthop. (2018) 15(2):366–71. 10.1016/j.jor.2018.01.039 29881155 PMC5990374

[15] Choi CM Chung JT Lee SJ Choi DJ. How I do it? Biportal endoscopic spinal surgery (BESS) for treatment of lumbar spinal stenosis. Acta Neurochir (Wien). (2016) 158(3):459–63. 10.1007/s00701-015-2670-7 26782827 PMC4752582

[16] Shen J Wang Q Wang Y Min N Wang L Wang F. Comparison between fusion and non-fusion surgery for lumbar spinal stenosis: a meta-analysis. Adv Ther. (2021) 38(3):1404–14. 10.1007/s12325-020-01604-7 33491158

[17] Weishaupt D Zanetti M Boos N Hodler J. MR Imaging and CT in osteoarthritis of the lumbar facet joints. Skeletal Radiol. (1999) 28(4):215–9. 10.1007/s002560050503 10384992

[18] Schreiber JJ Anderson PA Rosas HG Buchholz AL Au AG. Hounsfield units for assessing bone mineral density and strength: a tool for osteoporosis management. J Bone Joint Surg Am. (2011) 93(11):1057–63. 10.2106/JBJS.J.00160 21655899

[19] Courtois EC Ohnmeiss DD. Assessing bone quality in hounsfield units using computed tomography: what value should be used to classify bone as normal or osteoporotic? Eur Spine J. (2025) 34(2):493–7. 10.1007/s00586-024-08565-3 39576307

[20] Schizas C Theumann N Burn A Tansey R Wardlaw D Smith FW. Qualitative grading of severity of lumbar spinal stenosis based on the morphology of the dural sac on magnetic resonance images. Spine (Phila Pa 1976). (2010) 35(21):1919–24. 10.1097/BRS.0b013e3181d359bd 20671589

[21] Pfirrmann CW Metzdorf A Zanetti M Hodler J Boos N. Magnetic resonance classification of lumbar intervertebral disc degeneration. Spine (Phila Pa 1976). (2001) 26(17):1873–8. 10.1097/00007632-200109010-00011 11568697

[22] Zou D Li W Deng C Du G Xu N. The use of CT hounsfield unit values to identify the undiagnosed spinal osteoporosis in patients with lumbar degenerative diseases. Eur Spine J. (2019) 28(8):1758–66. 10.1007/s00586-018-5776-9 30306332

[23] Yang L Yu T Jiao J Hou T Wang Y Zhao B. Comprehensive analysis of UBE-related complications: prevention and management strategies from 4685 patients. Med Sci Monit. (2024) 30:e944018. 10.12659/MSM.944018 39385451 PMC11476038

[24] Lin G-X Huang P Kotheeranurak V Park C-W Heo D-H Park C-K. A systematic review of unilateral biportal endoscopic spinal surgery: preliminary clinical results and complications. World Neurosurg. (2019) 125:425–32. 10.1016/j.wneu.2019.02.038 30797907

[25] Lee MJ Hacquebord J Varshney A Cizik AM Bransford RJ Bellabarba C. Risk factors for medical complication after lumbar spine surgery: a multivariate analysis of 767 patients. Spine (Phila Pa 1976). (2011) 36(21):1801–6. 10.1097/brs.0b013e318219d28d 22046614 PMC3711415

[26] Alizadeh R Sharifzadeh SR. Pathogenesis, etiology and treatment of failed back surgery syndrome. Neurochirurgie. (2022) 68(4):426–31. 10.1016/j.neuchi.2021.09.005 34543614

[27] Schoenfeld AJ Ochoa LM Bader JO Belmont PJ. Risk factors for immediate postoperative complications and mortality following spine surgery: a study of 3475 patients from the national surgical quality improvement program. J Bone Joint Surg Am. (2011) 93(17):1577–82. 10.2106/JBJS.J.01048 21915571

[28] Alhaug OK Dolatowski FC Solberg TK Lønne G. Predictors for failure after surgery for lumbar spinal stenosis: a prospective observational study. Spine J. (2023) 23(2):261–70. 10.1016/j.spinee.2022.10.010 36343913

[29] Aimar E Iess G Mezza F Gaetani P Messina AL Todesca A. Complications of degenerative lumbar spondylolisthesis and stenosis surgery in patients over 80 s: comparative study with over 60 s and 70 s. Experience with 678 cases. Acta Neurochir (Wien). (2022) 164(3):923–31. 10.1007/s00701-022-05118-9 35138487 PMC8913488

[30] Nomeland AA Brekke EAM Seip AO Myklebust TÅ Ebbs EK Solberg T. Predictors for unsuccessful outcome of lumbar spinal stenosis surgery: a secondary analysis of the two randomized NORDSTEN trials. Spine (Phila Pa 1976). (2026) 51(8):527–33. 10.1097/BRS.0000000000005642 41626785

[31] Jiang W Zhao Y Du Y Cui K Yang C Zhao Z. Perioperative recognition and management of rare irrigation-related complications in unilateral biportal endoscopy under general anesthesia: two case reports. Eur Spine J. (2025) 34(12):5742–6. 10.1007/s00586-025-09077-4 40603648

[32] Friedman JA Ecker RD Piepgras DG Duke DA. Cerebellar hemorrhage after spinal surgery: report of two cases and literature review. Neurosurgery. (2002) 50(6):1361–4. 10.1097/00006123-200206000-00030 12015857

[33] You SH Son KR Lee NJ Suh JK. Remote cerebral and cerebellar hemorrhage after massive cerebrospinal fluid leakage. J Korean Neurosurg Soc. (2012) 51(4):240–3. 10.3340/jkns.2012.51.4.240 22737308 PMC3377885

[34] Basques BA Perez-Albela A Hanna J Knebel A Daher M Singh M. Postoperative foot drop after spinal surgery: etiology, presentation, and management strategies. JBJS Rev. (2025) 13(3). 10.2106/JBJS.RVW.24.00191 40153523

[35] Nath RK Somasundaram C. Incidence, etiology, and risk factors associated with foot drop. Eplasty. (2023) 23:e16. 37187871 PMC10176465

[36] Stewart JD. Foot drop: where, why and what to do? Pract Neurol. (2008) 8(3):158–69. 10.1136/jnnp.2008.149393 18502948

[37] Goyal VK Mathur V. Pé caído: uma complicação iatrogênica da anestesia espinhal [foot drop: an iatrogenic complication of spinal anesthesia]. Braz J Anesthesiol. (2018) 68(4):412–5. 10.1016/j.bjan.2017.09.007 29373140 PMC9391714

